# Case report: Therapeutic response to lorlatinib in advanced large-cell neuroendocrine carcinoma of the lung and breast cancer: a heterochronous double malignancy perspective

**DOI:** 10.3389/fphar.2024.1413897

**Published:** 2024-12-10

**Authors:** Xiaoli Mu, Yan Li

**Affiliations:** ^1^ The Department of Biotherapy, Cancer Center, West China Hospital, Sichuan University, Chengdu, Sichuan, China; ^2^ Lung Cancer Center, West China Hospital, Sichuan University, Chengdu, Sichuan, China

**Keywords:** large-cell neuroendocrine carcinoma, lorlatinib, multiple primary malignancies, ALK tyrosine kinase inhibitor (ALK-TKI), brain metastases

## Abstract

**Background:**

Driver mutations in tyrosine kinases, such as the anaplastic lymphoma kinase (ALK) mutation, are known to play a critical role in the pathogenesis of non-small cell lung cancer (NSCLC) but are rarely observed in large cell neuroendocrine carcinoma (LCNEC). Multiple primary malignancies (MPMs) refer to the occurrence of two or more distinct primary malignancies within the same or different organs and tissues in a single patient, either simultaneously or sequentially.

**Case Presentation:**

We reported a case of advanced LCNEC as a heterochronous double primary malignancy, following a prior breast cancer diagnosis in a 55-year-old woman. Ten years after achieving remission from breast cancer, the patient was diagnosed with LCNEC, presenting with multiple brain metastases (BMs) after undergoing surgery and adjuvant radiochemotherapy. She tested positive for the ALK fusion gene and received lorlatinib as an initial treatment. After 6 weeks, there was a significant reduction in the tumor, and the treatment impact was evaluated as a partial response. The treatment has been continued for over 25 months since the initiation of ALK Tyrosine kinase inhibitor (ALK-TKI) therapy.

**Conclusion:**

This case suggested that ALK-positive advanced LCNEC patients might benefit from first-line intervention with lorlatinib, particularly for managing brain metastases.

## Introduction

Multiple primary malignancies (MPMs) refer to the simultaneous occurrence of two or more distinct primary malignancies in the same or different organs in a single patient (Li et al., 2015; Irimie et al., 2010). Based on a 6-month diagnostic threshold, MPMs are classified as either synchronous or heterochronous. In a study of 1,104,269 cancer patients, the incidence of MPMs ranged between 0.73% and 11.7% (Demandante et al., 2003). According to the epidemiological survey and research of the Surveillance, Epidemiology, and End Results (SEER) database in the United States, the most common second primary cancers are lung, colorectal, prostate, and bladder cancers, with their incidence rates of 18%, 12%, 9% and 8%, respectively. Importantly, second primary cancers have been shown to be more lethal than initial primary cancers (Donin et al., 2016). Despite diagnostic criteria for MPMs existing since the late 19th century, no standardized treatment guidelines have been established to date.

Lung cancer remains one of the most prevalent cancers and has the highest mortality rate among all malignancies. Among the various subtypes, large cell neuroendocrine carcinoma (LCNEC) is rare but highly aggressive, associated with poor prognosis, with a median overall survival (OS) of only 8–12 months (Yang et al., 2022; Andrini et al., 2022). Given the rarity of LCNEC, most available evidence on LCNEC is based on small retrospective studies or extrapolated from treatment approaches for small cell lung cancer (SCLC) and non-small cell lung cancer (NSCLC) (Andrini et al., 2022). Clinical management of LCNEC remains challenging and controversial, with no standardized treatment strategies currently established.

ALK mutations are identified in 3%–7% of NSCLC patients (El Darsa et al., 2020). Although rare in LCNEC, ALK fusion genes have occasionally been detected (Zheng et al., 2018; Wang et al., 2017). The presence of ALK rearrangements is particularly significant, as it opens up possibilities for targeted therapy. Given the rarity of this mutation in LCNEC compared to NSCLC, its detection in LCNEC suggests a potential benefit from ALK-targeted treatments, warranting further exploration. ALK inhibitors like lorlatinib, a third-generation TKI with effective central nervous system (CNS) penetration, have shown substantial efficacy in managing ALK-positive NSCLC, especially in cases with brain metastases (BMs) (Tao et al., 2022).

In this report, we presented a case of heterochronous double primary malignancy involving breast cancer and ALK-positive advanced LCNEC. The successful use of lorlatinib, in this case, highlighted the therapeutic potential of ALK-targeted therapies for ALK-positive LCNEC, particularly in patients with BMs.

## Case presentation

The 55-year-old female patient underwent a modified radical mastectomy in July 2007. Pathological examination confirmed the presence of invasive ductal carcinoma in the left breast, classified as WHO grade III. Immunohistochemical analysis revealed positive expression of estrogen receptor (ER) and progesterone receptor (PR), focal positivity for PS2, and negativity for human epidermal growth factor receptor 2 (Her2). Additionally, the Ki67 proliferation index was approximately 20%. Metastatic spread was detected in the left axillary lymph nodes, with 7 out of 18 sampled showing involvement, and in the left subclavian lymph nodes, with 1 out of 2 sampled exhibiting metastasis. After mastectomy, the patient received adjuvant chemotherapy and radiotherapy directed at the left chest wall and supraclavicular area. Due to the hormone receptor-positive nature of the cancer, she underwent oophorectomy to reduce estrogen levels as a preventive measure against recurrence, followed by 5 years of endocrine therapy. Postoperative evaluations indicated no signs of residual lesions, confirming complete disease remission.

**FIGURE 1:**
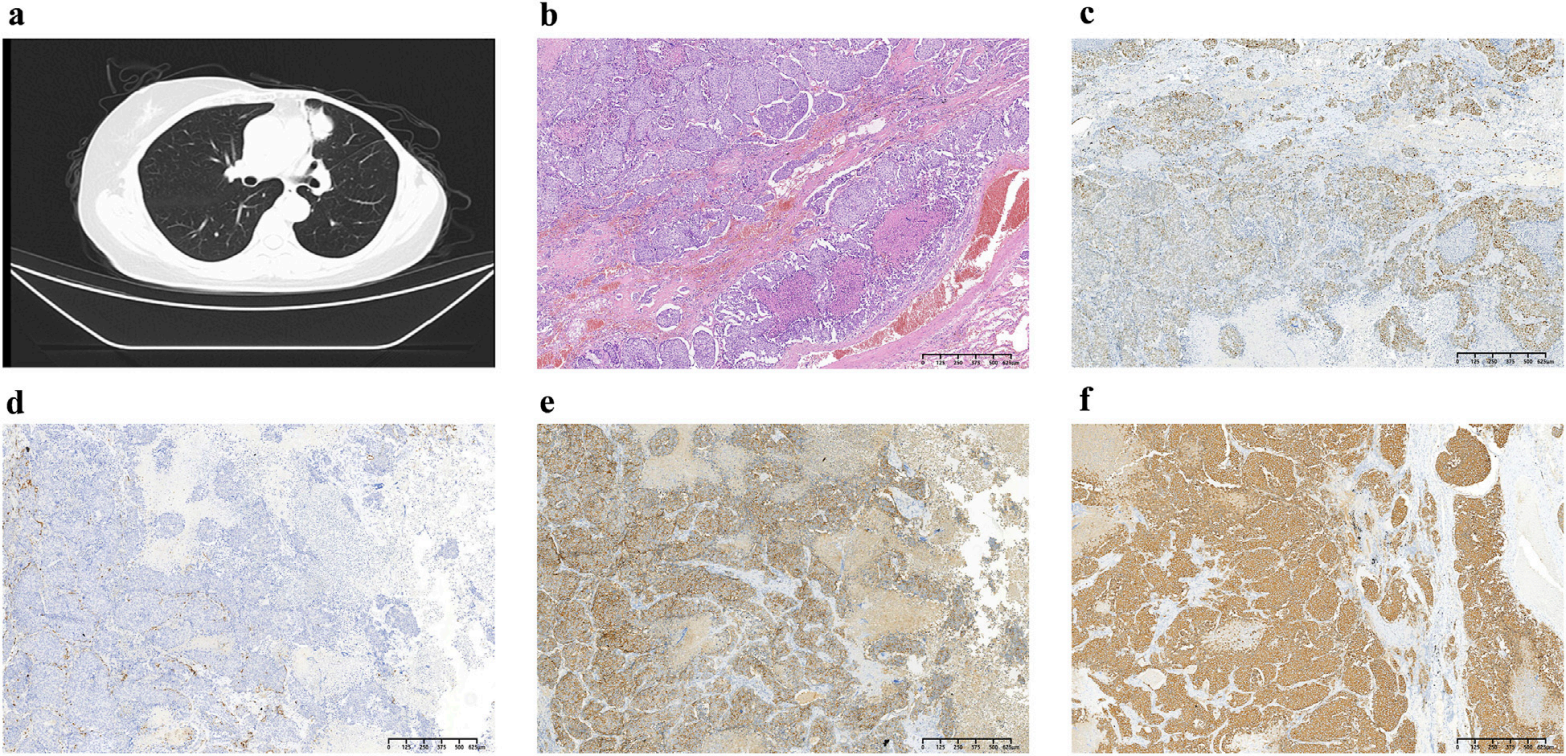
**(A)** Chest CT at diagnosis showing the presence of lung masses; **(B)** Postoperative pathology demonstrating hematoxylin and eosin (H&E) staining, which reveals diffuse small round cells with scant cytoplasm and inconspicuous nucleoli; **(C)** Immunohistochemical staining for TIF-1, indicating partial positivity (+); **(D)** Napsin A staining, which is negative (−); **(E)** CD56 staining showing positivity (+); **(F)** Synaptophysin staining demonstrating positivity (+). These results provide a comprehensive overview of the histological and immunohistochemical characteristics of the lung masses diagnosed.

**FIGURE 2:**
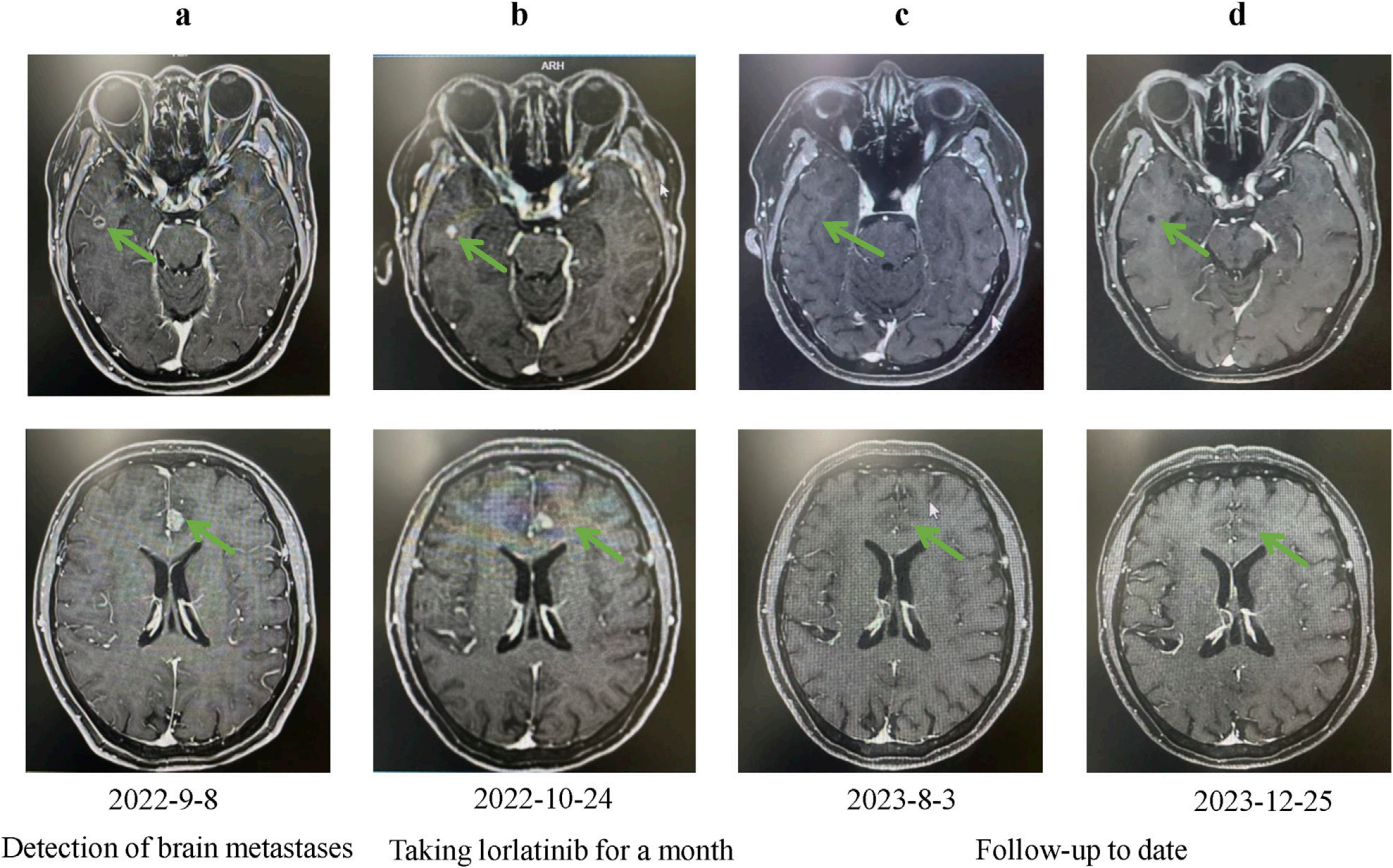
Changes in brain lesions during Lorlatinib treatment. **(A)** Baseline MRI showing brain lesions on 2022-9-8; **(B)** Follow-up MRI after one month of Lorlatinib treatment on 2022-10-24, showing partial reduction in lesion size; **(C)** Further improvement observed on MRI after sustained treatment on 2023-8-3; **(D)** Continued regression of brain lesions on MRI after long-term treatment on 2023-12-25.

**FIGURE 3:**
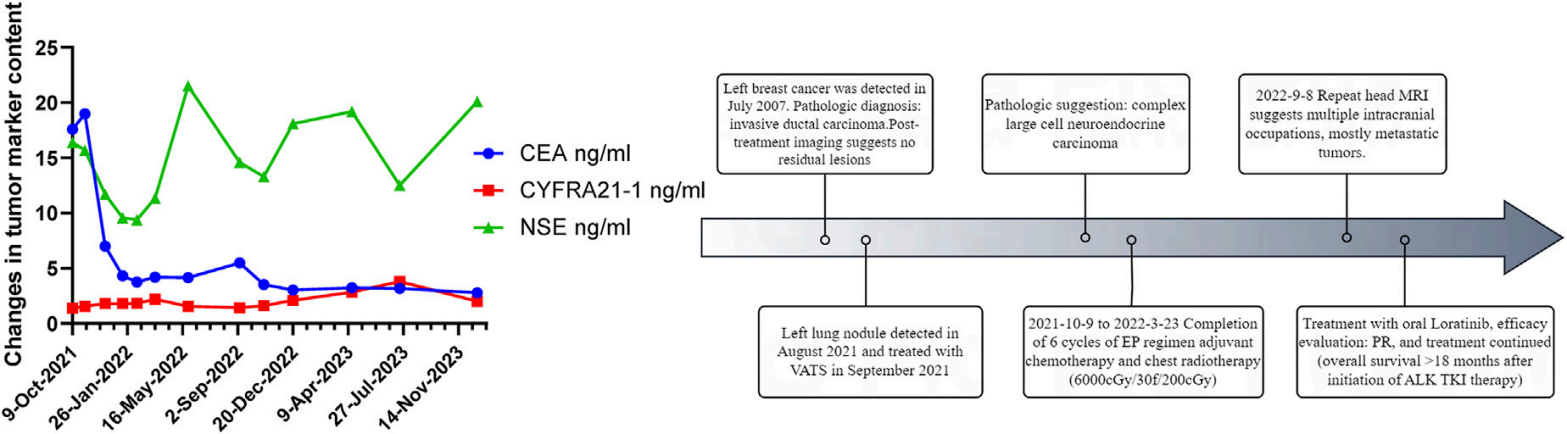
Clinical progression timeline of the patient.

In August 2021, 14 years after her breast cancer diagnosis, the patient was readmitted to the hospital after a physical examination revealed nodules in the upper left lung. She had no previous history of smoking; the computed tomography (CT) scan of the chest suggested a solid nodule in the upper lobe of the left lung, measuring about 3.3 × 2.4 cm in size (Figure 1A). Later, the patient underwent video-assisted thoracic surgery (VATS) with left upper lobectomy and lymph node dissection on 3 September 2021. Histological analysis revealed a complex LCNEC, comprising 97% LCNEC and 3% invasive adenocarcinoma (vesicular and minor solid type). Immunohistochemistry indicated that tumor cells were negative for Napsin A, partially positive for TTF-1, and positive for CD56 and synaptophysin (Figures 1B–F). Next-generation sequencing (NGS) confirmed an ALK-positive mutation. Lymphatic involvement was notable, with a substantial presence of cancer emboli detected in the pleura. Examination of lymph nodes revealed metastases in “group 5” (1 out of 1), “group 7” (1 out of 1), “group 10” (1 out of 1), and “group 12” (1 out of 1), along with involvement of peribronchial lymph nodes (2 out of 3). Consequently, the patient’s pathological stage was determined as pT2aN2M0, with a clinical stage corresponding to stage IIIA according to the American Joint Committee on Cancer (AJCC) 8th edition staging. Following surgery, the patient received six cycles of adjuvant chemotherapy (etoposide plus cisplatin) and chest radiotherapy (6,000 cGy/30 fractions). 11 months (2022-9-8) after receiving concurrent chemotherapy and radiation, the magnetic resonance imaging (MRI) of the brain revealed multiple new BMs, with the largest lesion located in the left frontal region near the cerebral falx, measuring approximately ×1.2 0.7 (Figure 2A). At this stage, potential treatment options included, 1. local radiotherapy/surgery, 2. systemic chemotherapy, and 3. oral ALK-TKI. It’s crucial to note that BMs can originate either from large neuroendocrine cells or from ALK-positive mutations. Surgical intervention proves to be challenging in such cases, particularly considering the multifocal nature of the disease. After thorough consideration, the patient chose lorlatinib because of its efficacy in targeting ALK-positive tumors involving the central nervous system. Given that both LCNEC and ALK-positive lung adenocarcinoma may present with multiple BMs, surgical resection alone can only confirm the diagnosis of a single tumor and is too invasive, while whole-brain radiotherapy is indicated only for palliative care. Loratinib was selected as the preferred first-line therapy over other systemic therapies due to its excellent blood-brain barrier permeability and high intracranial tumor control rate, providing a strategic approach to maximize intracranial efficacy with minimal systemic toxicity. In addition, we plan to evaluate the effect of lorlatinib on BMs after 1 month of treatment to determine the likely source of the metastases. If effective, this would indicate that the metastases were from ALK-positive lung adenocarcinoma; if not, they could be from LCNEC, leading to a timely shift to other treatments as needed. This approach ultimately proved successful, as 1 month after initiating lorlatinib (24 October 2022), the patient’s brain MRI showed partial disappearance and partial shrinkage of the intracranial nodules, with the left frontal parafoveal cerebral sickle nodule narrowing to 1.1 cm × 0.8 cm (Figure 2B), supporting our decision to use lorlatinib as the initial treatment strategy. Subsequent follow-up brain MRI in December 2023 revealed further shrinkage, with the left frontal lobe nodule measuring 0.6 × 0.4 cm and the right temporal lobe nodule nearly absent (Figures 2C, D). The patient remains in active treatment, with survival exceeding 25 months after the initiation of ALK-TKI therapy. Figure 3 illustrates the timeline of the patient’s treatment and changes in tumor marker.

## Discussion

Here, we reported a case of heterochronous double primary malignancy of breast cancer combined with ALK-positive advanced LCNEC, in which the patient experienced a beneficial response to ALK-TKI therapy following the failure of postoperative adjuvant therapy.

MPMs were first described as a case report by Billroth et al. back in 1889 (Billroth and von Winiwarter, 1889; Jiao et al., 2014). However, owing to the relative scarcity of its occurrences, the pathogenesis of MPMs remains to be determined. There are many possible reasons for the development of MPMs, including genetic susceptibility, intrinsic factors, bad lifestyle, and iatrogenic factors, including radiotherapy and chemotherapy (Sakellakis et al., 2014; Wood et al., 2012; Ye et al., 2022). Studies have indicated that Hodgkin lymphoma survivors who received chest radiotherapy face a higher risk of developing lung cancer compared to those who did not (Tucker et al., 1988; Travis et al., 2002). Similar findings have been reported in female patients who have received radiotherapy for breast cancer (Kaufman et al., 2008). The incidence of lung cancer was significantly higher in women with a history of smoking who received radiotherapy after mastectomy for breast cancer compared with women who had never smoked and did not receive post-mastectomy radiotherapy. The patient, in this case, was diagnosed with a heterochronous double primary malignancy comprising breast cancer and advanced composite LCNEC. She had previously undergone chest wall radiotherapy, which might have contributed to the development of the double cancer.

LCNEC is a rare but highly aggressive neuroendocrine differentiated tumor that accounts for approximately 3% of all lung cancers (Asamura et al., 2006). ALK rearrangements are commonly identified in lung adenocarcinoma; LCNEC with ALK rearrangements is exceedingly rare and often associath-grade pathology, advanced-stage presentation, and poor prognosis (Zheng et al., 2018; Shaw et al., 2013). This case was pathologically diagnosed as complex LCNEC, containing a minor (3%) adenocarcinent. Lorlatinib, a brain-penetrant, third-generation ALK and ROS1 tyrosine kinase inhibitor, is widely used for ALK-positive tumors. In November 2018, the FDA approved lorlatinib as a treatment option for ALK-positive metastatic NSCLC in the second or third line (Solomon et al., 2018). Following that, in March 2021, it received approval for use as a first-line treatment for ALK-positive advanced NSCLC (Nagasaka and Ou, 2021). There is a high incidence of BMs in patients with ALK-positive NSCLC, with initial BMs occurring in 15%–35% of cases, increasing to 60% during first-line TKI or chemotherapy treatment (Shaw et al., 2013; Doebele et al., 2012; Guerin et al., 2015).

In a multicenter phase II study, lorlatinib has shown substantial overall and intracranial activity both in the first-line and subsequent treatment of patients with ALK-positive NSCLC (Solomon et al., 2018). A recent meta-analysis confirmed that lorlatinib was superior to other ALK inhibitors such as brigatinib, alectinib, ceritinib, crizotinib, and chemotherapy in prolonging progression-free survival (PFS) (Ando et al., 2023). Notably, lorlatinib’s blood-brain solid barrier penetration and its efficacy in CNS metastases have positioned it as the most effective third-generation ALK inhibitor for managing intracranial lesions, especially in comparison to second-generation inhibitors, which are comparatively less effective in CNS control (Ando et al., 2023; Solomon et al., 2023). The CROWN study—a phase III clinical trial—reported lorlatinib’s significant CNS efficacy. With over 5 years of follow-up, lorlatinib achieved the longest reported PFS in advanced ALK-positive NSCLC, with a PFS rate of 60%. Among patients without baseline BM, the 5-year intracranial progression-free rate reached 96%, and the hazard ratio (HR) was as low as 0.05, reflecting a 95% reduction in the risk of disease progression and mortality. In patients with measurable BM at baseline, lorlatinib achieved an intracranial objective response rate (ORR) of 92%, making it the only ALK-TKI to achieve remission of intracranial lesions in over 90% of patients. Additionally, lorlatinib’s intracranial Complete Response (CR) rate reached 58%, indicating that it is currently the only ALK-TKI capable of eliminating intracranial lesions in nearly 60% of patients (Solomon et al., 2023; Solomon et al., 2024).

To the best of our knowledge, there were a limited number of prior case reports detailing ALK-positive LCNEC patients, most of whom were treated with sequential ALK inhibitors and and only started using lorlatinib once they became TKI-resistant experienced disease progression (Leblanc et al., 2021; Akhoundova et al., 2022; Wiedemann et al., 2022). A recent case report of a pulmonary ALK-positive LCNEC demonstrated sustained clinical benefit (OS 24+ month) after sequential ALK inhibitors and local therapy, however, the patient progressed after 9 months on Alectinib, and brain metastatic lesions increased again after 4 months of switching to Brigatinib (Kobayashi et al., 2023). Similarly, most reported cases of ALK-positive LCNEC BMs in the literature have been treated with first-line crizotinib or alectinib, suggesting a need for more effective CNS-targeted options.

In the case of neuroendocrine large-cell carcinoma, treatment is tailored based on the degree of malignancy. It is worth mentioning that in this study, the EC chemotherapy regimen specifically targeted the more aggressive neuroendocrine large-cell carcinoma component, effectively suppressing the dominant subclone. However, as the EC regimen did not address the ALK-positive adenocarcinoma component, there was a risk that the ALK-positive adenocarcinoma could grow and metastasize as a non-dominant subclone, warranting an ALK-targeted approach to prevent progression.

In this case, the treatment focus was on selecting an effective yet minimally invasive approach, with lorlatinib emerging as a viable choice due to its superior CNS penetration and efficacy in ALK-positive tumors with BMs. This report was the first to demonstrate lorlatinib as a first-line therapeutic agent for advanced LCNEC, showing significant survival benefits. The patient, currently under active treatment, has achieved a PFS of over 25 months, underscoring the potential of lorlatinib in prolonging survival in similar cases.

The positive response in this case suggestted that ALK-TKIs like lorlatinib could be beneficial for other ALK-positive LCNEC patients, especially those with CNS involvement. However, considering the rarity of ALK mutations in LCNEC, lorlatinib may be best reserved for cases with confirmed ALK-positivity and CNS metastases, pending further research to establish broader clinical guidelines.

## Conclusion

Lorlatinib is active in the treatment of CNS metastases for ALK-positive advanced LCNEC.The significantly improved PFS observed in this patient emphasizes the importance of lorlatinib as the first-line treatment for advanced LCNEC.

## Data Availability

The original contributions presented in the study are included in the article/supplementary material, further inquiries can be directed to the corresponding author.
